# Fetal Toxicity of Immunosuppressive Drugs in Pregnancy

**DOI:** 10.3390/jcm7120552

**Published:** 2018-12-15

**Authors:** Claudio Ponticelli, Gabriella Moroni

**Affiliations:** 1 Former Director Renal Unit, Fondazione IRCCS Ca’ Granda Ospedale Maggiore Policlinico, 20122 Milan, Italy; 2 Nephrological Unit, Fondazione IRCCS Ca’ Granda Ospedale Maggiore Policlinico, 20122 Milan, Italy; gabriella.moroni@policlinico.mi.it

**Keywords:** pregnancy, autoimmune diseases, organ transplantation, neoplasia

## Abstract

Women affected by autoimmune diseases, organ transplantation, or neoplasia need to continue immunosuppressive treatment during pregnancy. In this setting, not only a careful planning of pregnancy, but also the choice of drugs is critical to preventing maternal complications and minimizing the fetal risks. Some immunosuppressive drugs are teratogenic and should be replaced even before the pregnancy, while other drugs need to be managed with caution to prevent fetal risks, including miscarriage, intrauterine growth restriction, prematurity, and low birth weight. In particular, the increasing use of biologic agents raises the question of their compatibility with reproduction. In this review we present data on the indication and safety in pregnancy of the most frequently used immunosuppressive drugs.

## 1. Introduction

Today pregnancy is not considered as a formal contraindication for women with autoimmune disease, organ transplantation, or even neoplasia as long as it is carefully planned. Yet, the need of continuing immunosuppressive drugs in these patients represents a major issue because of the potential teratogenic or toxic effects on the fetus. In this review we report the possible adverse events on the fetus and newborn of the immunosuppressive agents currently used in autoimmune diseases, organ transplantation and neoplasia. In order to analyze the problems physicians face with the use of immunosuppressive drugs during pregnancy, we performed an electronic search throughout the PubMed literature for articles published from 1 January 1990 through 1 October 2018, using the keywords *pregnancy, teratogenic drugs, drug (glucocorticoids, calcineurin inhibitors etc.) and pregnancy, fetal toxicity, selecting those presenting original data. Most articles reported the results of observational studies with a small number of participants. We mainly considered large reports and studies of comparison with other drugs*.

## 2. Glucocorticoids

Glucocorticoids may exert their activities by two main mechanisms of action: the classic genomic effects, that require the translocation to the nucleus of the complex glucocorticoid-glucocorticoid receptor, and secondary nongenomic effects, that are probably mediated by plasma membrane receptors. Synthetic glucocorticoids are powerful anti-inflammatory agents and can also interfere with the immune response, cellular immunity being more susceptible than humoral immunity. These and other actions which involve most cells of the body, occur simultaneously and are mainly mediated by the inhibition of the proinflammatory transcription factors nuclear factor of kinase B and activator protein-1. Glucocorticoids are largely used in most autoimmune diseases, oncology, and organ transplantation.

In pregnant women glucocorticoids may cause a predisposition to hypertension and preeclampsia when used at high doses. These agents easily cross the placenta [1], but 90% of the maternal dose of glucocorticoids is metabolized within the placenta by 11β-hydroxysteroid dehydrogenase-2 (11β-HSD2) which converts cortisol, prednisone, and methylprednisolone into inactive products while dexamethasone and betamethasone are less well metabolized [2,3]. There has been concern about a possible increase of oral-facial clefts in newborns from mothers receiving glucocorticoids. This risk is not strong, being approximately 1.3 to 3.3 for every 1000 pregnancies exposed to glucocorticoids during the critical period versus a birth population prevalence of 1 per 1.000 [4]. Further studies confirmed that the risk of oral-facial cleft associated with prenatal exposure to glucocorticoids is minimal [5]. It has been reported that cortisol exposure at earlier gestations can influence fetal growth [6,7] and that high levels of maternal endogenous glucocorticoids, or treatment with exogenous glucocorticoids, may result in dysfunction of the fetal hypothalamic–pituitary–adrenal axis (HPA) with permanent changes in physiology, structure and metabolism that might cause a number of chronic diseases in later life [8,9]. This phenomenon, termed early life programming, can persist throughout the life of an organism and may be associated in the long-term with impaired brain growth, altered behavior, and increased susceptibility to metabolic and cardiovascular disease. However, since only 3% of maternal cortisol is transferred to the fetal circulation [10], it is unlikely that moderate doses of exogenous glucocorticoids may interfere with the early life programming, unless there are alterations of 11β-HSD2 activities, as in the case of pre-eclampsia [11]. At any rate, the potential risks of prednisone and analogues should not prevent dexamethasone or betamethasone administration for fetal lung maturation in cases of threatened preterm labor. The Food and Drug administration (FDA) classifies the fetal risk of glucocorticoids in the category C, meaning that human risk cannot be ruled out (Table 1). 

In summary, the risk of malformation in newborns from mothers taking glucocorticoids is low. Also, the risk of fetal HPA dysfunction is questionable. However concern remains about the fetal toxicity when the mothers receive prolonged treatments with high-dose glucocorticoids or develop pre-eclampsia. We feel that doses of prednisone (or equivalent agents) <20 mg daily may be considered safe for the mother and newborn [12]. Prednisone is compatible with breastfeeding.

## 3. Calcineurin Inhibitors (CNIs)

The two principal CNIs are cyclosporine (CsA) and tacrolimus (TAC). They have different formulas and metabolism but a similar mechanism of action. CNI exert their actions through the inhibition of a calcium-dependent serum/threonine phosphatase, called calcineurin. Circulating CNIs easily enter the cell membrane. Within cells, CNI binds to a specific protein receptor, called cyclophilin for CsA and FK-binding protein 12 for TAC. The complex CNI receptor binds to and inhibits calcineurin, which has a key role in T-cell activation [13]. Calcineurin removes phosphates from a family of transcription factors called nuclear factors of activated T cells, therefore allowing them to enter the nucleus and collaborate to the synthesis of interleukin-2, which has critical roles in key functions of the immune system, tolerance, and immunity, primarily via its direct effects on T cells [14]. As a consequence of calcineurin inhibition the proliferation and differentiation of cytotoxic and other effector T cells are inhibited. Both CNIs can induce renal fibrosis that has been attributed to an exaggerated deposition of extracellular matrix, due to an increased expression of transforming-growth factor beta [15]. CNIs are the cornerstone of immunosuppressive therapy in organ transplantation and are also largely used in autoimmune diseases.

### 3.1. Cyclosporine

Animal studies have shown reproductive toxicity manifested by increased pre- and postnatal mortality. Reduced fetal weight, and skeletal retardation, low birth weight, cesarean delivery, and hypertensive disorders of pregnancy have been frequently reported in pregnant transplanted women. However, it is difficult to identify the role of CsA since transplant recipients are treated with a number of drugs, including other immunosuppressant agents. CsA is very lipid-soluble drug, is extensively distributed in the body, and is highly metabolized. High concentrations of CsA metabolites in the placenta can be observed, indicating the presence of CsA metabolizing enzymes in the placenta [16]. The maternal–fetal trans-placental passage of CsA can be influenced by the functional activity of P-glycoprotein. In an experimental model, it was demonstrated that P-glycoprotein pumps CsA out of the trophoblast cells of the rat placenta in the ATP-dependent manner and restricts the passage of CsA across the placental barrier [17]. The drug does not appear to be teratogenic [18,19,20]. The FDA classifies CsA as category C, meaning that human risk cannot be ruled out. Theoretically, calcineurin inhibitors might alter the immune status of the infant; however, no reports were found. On the other hand, some investigators found that low-dose CsA treatment can regulate the immune response and increase the live birth rate in mothers with unexplained recurrent miscarriage [21]. There has been concern about the effects of the child’s exposure to CsA excreted into the breast milk. However, no lingering effects due to breast-feeding have been found in infants who were breast-fed while their mothers were taking CsA [22,23].

### 3.2. Tacrolimus

TAC crosses the placenta with in utero exposure being approximately 71% of maternal blood concentrations [24] TAC metabolism by the fetus is limited, probably because the most abundant cytochrome enzyme in the human liver during fetal stages is CYP3A7, which shows a large interindividual variability of an unknown molecular basis [25]. The lower fetal blood concentrations are likely due to active efflux transport of TAC from the fetus toward the mother by placental P-glycoprotein [24]. In newborns from transplant recipients the risk of major malformations was low. As with CsA there is an increased risk of low birth weight and *preterm* birth [26,27]. Both CNIs can cause reversible nephrotoxicity and hyperkalemia in the newborn [24]. Ingestion of TAC by infants via breast milk is negligible. Breastfeeding does not appear to slow the decline of infant TAC levels from higher levels present at birth. Women taking TAC should not be discouraged from breastfeeding if monitoring of infant levels is available [28,29].

In summary, in CNI-treated pregnant women the maternal–fetal outcomes mainly depend on the maternal conditions and are not particularly influenced by the use of CNI. This seems to be confirmed by large reviews of pregnant transplanted women taking CNI. In the studies, the maternal-fetal outcomes of transplanted patients were comparable with those of non-transplanted patients with similar levels of kidney function impairment [30]. The relatively high number of premature births that has been reported by some series may be partially explained by an obstetric policy favoring earlier delivery [31]. Nonetheless, long-term effects in humans prenatally exposed to CNIs require further evaluation.

## 4. Nucleotide Synthesis Inhibitors

Activated T lymphocytes require the synthesis of nucleotides to proliferate and differentiate into T cell effectors. There are two main categories of antiproliferative agents that may inhibit the nucleotide synthesis: azathioprine and mycophenolate salts. These drugs are extensively used in autoimmune diseases and organ transplantation often in association with glucocorticoids and/or a calcineurin inhibitor.

### 4.1. Azathioprine

Azathioprine is an orally absorbed prodrug, being a modification of 6-mercaptopurine, which is in turn an analogue of the purine basis hypoxanthine. After oral administration, azathioprine is rapidly transformed into 6-mercaptopurine by hepatic and erythrocyte glutathione. Mercaptopurine is bio-transformed into mercaptopurine nucleotides (thioinosine monophosphate, thioguanine and 6-thioguanine nucleotides) that inhibit the synthesis and utilization of precursors of RNA and DNA, so halting the proliferation of activated lymphocytes. Thioguanine nucleotides are incorporated into human bone marrow cells. Leucopenia is the most common manifestation of bone marrow toxicity, thrombocytopenia and megaloblastic anemia may also occur. There are two main pathways of degradation: direct oxidation by the enzyme xanthine oxidase and S-methylation. Only inactive metabolites pass into the fetal circulation.

The FDA classified azathioprine as a drug at potential risk of teratogenic effects [Class C) based on animal studies. However, clinical experience did not show an excess of malformations in women exposed to azathioprine during pregnancy. In a large retrospective study, 178 pregnancies in 172 women with systemic lupus erythematosus (SLE) were investigated. Of them, 87 were exposed to azathioprine and 91 were not. The use of other drugs was similar in both groups. The rate of live births, spontaneous abortions, weeks of gestation, rate of birth weight <2500 g, and low birth weight at term did not differ between the two groups. No infant had major congenital abnormalities [32]. Using the French pregnancy database a prospective comparative study reported that first trimester exposure to azathioprine in 124 pregnancies was not associated with an elevated rate of birth defects [33]. Also in organ transplanted women, the use of azathioprine during pregnancy is considered to be relatively safe [30,34,35,36].

In summary, azathioprine is considered safer than other drugs, such as mycophenolate or alkylating agents, and can be used to replace them in pregnant women with autoimmune disease or organ transplantation. Of some concern, however, older studies reported a rise of sister chromatid exchange frequency in patients treated with azathioprine [37,38]. Whether such a mild chromosome damage may have long-term consequences in the mothers and their newborns is unknown at present.

### 4.2. Mycophenolate Salts

There are two types of mycophenolate salts: mycophenolate mofetil and mycophenolate sodium. Both of them are prodrugs that release mycophenolic acid (MPA), an inhibitor of inosine-5′-monophosphate dehydrogenase, an enzyme essential for *de novo* purine synthesis. MPA particularly affects T and B lymphocytes since they rely almost exclusively on *de novo* purine synthesis. Use of mycophenolate drugs during pregnancy is associated with an increased risk of first trimester pregnancy loss and increased risk of congenital malformations, especially external ear and other facial abnormalities including cleft lip and palate, and anomalies of the distal limbs, heart, esophagus, and kidney [39,40,41]. The FDA classifies these drugs as Category D., i.e., with positive evidence of human fetal risk. It is recommended that pregnancy be avoided by women taking MPA [42]. Women being considered for treatment with MPA should always have a negative pregnancy test and should employ at least two methods of contraception during its use [43,44]. A retrospective cohort study investigated the association between the discontinuation of MPA and pregnancy after kidney transplantation in 382 cases. Birth defects and miscarriages were similar among patients who discontinued MPA >6 and <6 weeks prior to pregnancy and during the first trimester. In contrast, discontinuing MPA during the second trimester or later significantly increased the risk of miscarriages (odds ratio 9.35) and birth defects (odds ratio 6.06). The study concluded that it is beneficial for the fetus to discontinue Mycophenolate at any time prior to the second trimester [45].

In summary, if pregnancy occurs mycophenolate should be stopped as early as possible and replaced by azathioprine. The later mycophenolate is discontinued the higher the risk of complications. However, paternal exposure to mycophenolate did not increase the risk of adverse birth outcomes in children fathered by male kidney transplanted patients. These data support the continuation of paternal mycophenolate treatment before, during, and after conception [46]. Little information is available on the long-term effects in infants born to mothers who had been exposed to mycophenolate.

### 4.3. Leflunomide

Leflunomide is an isoxazole derivative, a prodrug that releases the active compound A771726. This achieves its immunosuppressive effects by inhibiting the mitochondrial enzyme dihydroorotate dehydrogenase. This enzyme plays a key role in the *de novo* synthesis of uridine monophosphate, which is required for the synthesis of DNA and RNA. Leflunomide is mainly used in rheumatoid arthritis. It has also been used to treat polyomavirus BK infection in immunosuppressed patients.

Animal studies indicate that the exposure to leflunomide during pregnancy has teratogenic and fetotoxic effects. Leflunomide has been classified as pregnancy category X by the FDA. The manufacturer recommends that for women of childbearing age treatment with leflunomide must not be started until pregnancy is excluded and that reliable contraception is being used [47]. Since leflunomide metabolites are detectable in maternal plasma for a long period, pregnancy should be programmed at least 2 years after discontinuation of leflunomide [48]. However, some data may provide reassurance to women who inadvertently become pregnant while taking leflunomide and undergo the washout procedure. In a prospective cohort study, 64 pregnant women with rheumatoid arthritis who were treated with leflunomide during pregnancy (95.3% of whom also received cholestyramine) were compared with 108 pregnant women with rheumatoid arthritis not treated with leflunomide, and 78 healthy pregnant women. There were no significant differences in the overall rate of major structural defects in the exposed group (3 of 56 live births, or 5.4%) relative to either comparison group (each 4.2%). The rate was similar to the 3–4% expected in the general population. There was no specific pattern of major or minor anomalies. Infants in both the leflunomide-exposed and non-leflunomide-exposed rheumatoid arthritis groups were born smaller and earlier relative to infants of healthy mothers. However, after adjustment for confounding factors, there were no significant differences between the leflunomide-exposed and non-leflunomide-exposed groups [49]. Other small series reported that maternal exposure to leflunomide during pregnancy was not associated with statistically significant increased risk of prematurity, low body weight, or miscarriages [50,51].

In spite of a few anecdotal cases of successful pregnancy in women exposed to leflunomide the risk of fetal toxicity remains elevated and leflunomide administration should be interrupted at least 2 years before a programmed pregnancy. In the case of unplanned pregnancy, the use of leflunomide should be stopped as soon as possible.

## 5. Alkylating Agents

Alkylating drugs have the capacity to contribute alkyl groups to DNA, so inducing inhibition of DNA replication and cell death. These drugs, in particular cyclophosphamide, are largely used as a chemotherapeutic agents in oncology but also in autoimmune diseases since they can also exert immunosuppressive activity by causing cytotoxic effects on proliferating lymphocytes.

### 5.1. Cyclophosphamide

The drug is converted by the enzymes of cytochrome P450 system to 4-hydroxycyclophosphamide which is in equilibrium with its tautomer aldophosphamide. This is oxidized by aldehyde dehydrogenase to inactive carboxycyclophosphamide, but some amount of aldophosphamide escapes the effects of aldehyde dehydrogenase and is cleaved to two toxic metabolites: the alkylating phosphoramide mustard and the teratogenic acrolein [52].

Miscarriages and pre-term deliveries have been reported in mothers taking cyclophosphamide [52,53,54]. Teratogenicity of cyclophosphamide is well demonstrated in animals. Cases of malformation have been described in newborns of mothers who received cyclophosphamide in the first months of pregnancy [55,56,57,58]. The drug is contraindicated in pregnancy (FDA Class D). All women contemplating its use must have a negative pregnancy test before starting the drug and use at least two methods of contraception. However, in a cohort of 81 pregnant women with breast cancer who received cyclophosphamide during the second and third trimester the rate of congenital abnormalities was similar to the national average of 3% [59]. At least in rats, diallyl disulfide may induce CYP3A1 expression in the placenta and exert potent antioxidant effects that attenuate the fetal toxicity of cyclophosphamide [60]. Instead, inducers of cytochrome P450 enzymes, such as green tea or licorice, may increase cyclophosphamide teratogenicity [61,62].

### 5.2. Chlorambucil

Chlorambucil alkylates and cross-links DNA during all phases of the cell cycle. Its interference with DNA replication damages the DNA in a cell and induces cell cycle arrest and cellular apoptosis.

The drug is mutagenic (FDA Category D) and can cause urogenital malformations including unilateral renal agenesis. The gene mutations induced by chlorambucil are dose-dependent and cumulative and the drug is contraindicated in pregnancy, particularly in the first trimester [63,64,65].

In summary, cyclophosphamide and chlorambucil should be discontinued for at least 3 months before conception and are contraindicated in the early pregnancy. In life-threatening situations the alkylating agent can be used in the second or third trimester [54]. There are few data on the long-term outcomes of children exposed to chemotherapy *in utero*. In one study, 84 children who were born to mothers who received chemotherapy (including cyclophosphamide) during pregnancy were examined after a median follow-up of 18.7 years. No congenital, neurological, or psychological abnormalities were observed. No cases of cancer or acute leukemia developed [66].

## 6. Mammalian Target of Rapamycin (mTOR) Inhibitors

Two mTOR inhibitors with a similar chemical composition are commercially available: rapamycin (sirolimus), a macrocyclic lactone of fungal origin, and everolimus in which a covalently bound 2-hydroxyethyl group was introduced at position 40. After oral administration, the mTOR inhibitors enter the cells and bind to a specific cytoplasmic receptor, an immunophilin called FK binding protein12 that also serves as a receptor of tacrolimus. The complex drug-receptor inhibits mTOR, the downstream effector of a family of kinases originated by phosphatidylinositol 3-kinase which, together with a protein kinase B and through the mediation of mTOR, activates a cascade of kinases that originate several signaling pathways necessary for T cell proliferation [67]. The mTOR inhibitors are mainly used for the immunosuppressive therapy in organ transplantation.

mTOR inhibitors increase the mortality of fetus in experimental animals. However, no teratogenic effects have been seen either in rats or rabbits. There is insufficient information about pregnant women treated with these drugs. Anecdotal cases of healthy newborns from mothers receiving mTOR inhibitors have been reported [68,69,70,71]. The FDA classifies these drugs as category C (teratogenic risk cannot be ruled out because of lack of information).

## 7. Monoclonal Antibodies

### 7.1. Rituximab (RTX)

RTX is a chimeric human/murine monoclonal antibody with a high affinity for the CD20 antigen, a membrane protein expressed on B cells. RTX induces a very rapid elimination of circulating B cells (measured in hours) that may be maintained for weeks or months. A large body of evidence shows that RTX depletes CD20+ B cells through three possible mechanisms of action: antibody-dependent cell-mediated cytotoxicity, cell-mediated cytotoxicity, and apoptosis. RTX is used for non-Hodgkin’s lymphoma, chronic lymphocytic leukemia, and in most autoimmune diseases.

According to the FDA a teratogenic risk cannot be excluded for RTX (category C). However, despite counselling to avoid pregnancy, women may inadvertently become pregnant during or after RTX treatment. In a retrospective study, 231 pregnancies associated with maternal RTX exposure were evaluated. Of 153 pregnancies with known outcomes, 90 resulted in live births. The high rate of miscarriages (22%) may be attributed to concomitant therapy for malignant or autoimmune disease. Twenty-two infants were born prematurely; with one neonatal death at 6 weeks. Eleven neonates had hematologic abnormalities; none had corresponding infections. Four neonatal infections were reported. Two congenital malformations were identified: clubfoot in one twin, and cardiac malformation in a singleton birth. One maternal death from pre-existing autoimmune thrombocytopenia occurred. A reversible B cell deficiency was noted in infants born of mothers who received RTX during the second and the third trimester of pregnancy [72]. According to the present counselling, women should avoid pregnancy for about 6 months after RTX exposure. The administration of RTX to a pregnant woman should be discouraged unless the benefits outweigh the potential risk for the fetus [73,74,75]. There are no data on RTX use in breastfeeding. The long-term effects of in utero exposure to RTX are unknown.

### 7.2. Eculizumab

Eculizumab is a fully humanized monoclonal antibody directed against the complement protein C5. By binding to C5 with high affinity, eculizumab inhibits its cleavage to C5a and C5b and prevents the generation of the inflammatory terminal complement complex C5b-9 (also called membrane-attack complex) which exerts hemolytic activity. Eculizumab preserves the early components of complement activation that are essential for opsonization of microorganisms and clearance of immune complexes. The main indications for eculizumab are paroxysmal hemoglobinuria nocturnal (PNH), atypical hemolytic uremic syndrome and C3 glomerulopathy. It has also been used for treating antibody-mediated rejection of organ transplantation.

In animals, eculizumab crosses the placenta and may cause increased rates of development abnormalities and mortality. Rare cases of retinal dysplasia have been reported in newborns from mothers treated with Eculizumab. There is little experience with the use of eculizumab in pregnant women. Most data come from pregnancies in women with PNH. To assess the safety and efficacy of eculizumab in pregnant women with PNH a questionnaire was sent to physicians participating to the international PNH registry. Data on 75 pregnancies in 61 women were evaluated. There were no maternal deaths and three fetal deaths (4%). Six miscarriages (8%) occurred during the first trimester. A total of 25 babies were breast-fed, and in 10 of these cases, breast milk was examined for the presence of eculizumab; the drug was not detected in any of the 10 breast-milk samples. The investigators concluded that eculizumab provided benefit for women with PNH during pregnancy, as evidenced by a high rate of fetal survival and a low rate of maternal complications [76]. Similar conclusions were reported by a multicenter Japanese study [77]. Theoretically, eculizumab may also cause terminal complement inhibition in the fetal circulation. However, in a study on two newborns from mothers treated with eculizumab during pregnancy, the antibody neither accumulated in fetal plasma nor impaired the complement function in the newborn [78]. However, according to the manufacturer, the drug should be administered to pregnant women only if benefits may justify the potentially increased risk for the fetus. Women of childbearing potential have to use effective contraception during treatment and up to 5 months after treatment.

### 7.3. TNF-Inhibitors

Infliximab, adalimumab, golimumab, and certolizumab pegol are monoclonal antibodies directed against tumor necrosis factor α (TNFα) but not TNFβ. These drugs are approved by regulatory authorities for treatment of rheumatoid arthritis, ankylosing spondylitis, plaque psoriasis, psoriatic arthritis. Adalimumab and certolizumab are administered by subcutaneous injection, Infliximab is injected intravenously and golimumab may be administered subcutaneously or intravenously.

The available data about pregnancy mainly comes from case-reports or retrospective studies. In those studies, there was no excess of birth defects with Infliximab. However, exposed newborns were more likely to be born prematurely and to be lower in body weight than other newborns from mothers with rheumatoid arthritis [79,80,81,82]. A recent report analyzed data from 1457 pregnancies exposed to infliximab or adalimumab, with 1313/7722 (17.0%) suffering from Crohn’s disease and 144/3553 (4.1%) from ulcerative colitis. After adjusting for a number of other variables, anti-TNFα treatment was associated with a higher risk of overall maternal complications (Odds ratio 1.49) and infections (Odds ratio 1.49 and 1.31 respectively). Maintaining anti-TNFα after 24 weeks did not increase the risk of maternal complications, but interrupting the anti-TNFα increased relapse risk. If these drugs are continued later in pregnancy, live vaccines should be avoided in the newborns until 7 months of age. No increased risk for infection was found in children up to one year of life [83]. The FDA includes infliximab and adalimumab in the category B (no documented increased risk for structural defects). The British Society of Rheumatology suggests that infliximab may be continued until 16 weeks and adalimumab may be continued until the second trimester of pregnancy when strictly indicated [84].

There is limited information about the use of golimumab in pregnancy. Lau et al. [85] reviewed 42 pregnant women exposed to golimumab. These pregnancies resulted in 19 live births, 13 spontaneous abortions, and 6 elective abortions. Of the 13 mothers who experienced miscarriages, 30.8% received simultaneous methotrexate treatment. Because golimumab is a large protein molecule, the amount in milk is likely to be very low. The FDA classifies intravenous golimumab in category B.

Certolizumab pegol has minimal to no active placental transfer. Data extracted from the UCB Pharma safety database showed that of 538 pregnancies exposed to Certolizumab, 459 resulted in live births (85.3%), 47 in miscarriages (8.7%), 27 in elective abortions (5.0%), and 5 in stillbirths (0.9%). There were 8 major congenital malformations (1.7%) among the 459 infants [86]. The data are reassuring for women of childbearing age considering treatment with certolizumab pegol.

### 7.4. Belimumab

Belimumab is a fully humanized monoclonal antibody that specifically binds to the soluble B lymphocyte stimulator (BlyS) causing a reduction in the number of peripheral naïve, transitional and activated B cells. It was approved in 2011 for systemic lupus erythematosus treatment and for this reason the experience with pregnancy is still poor. In pregnant monkeys treated with intravenous belimumab throughout gestation, transplacental passage was demonstrated, but no congenital malformation was observed in newborns [87]. Only 95 cases of pregnancies in women receiving belimumab in controlled randomized trials have been reported from GlaxoSmithKline. Most of these patients were receiving concomitant medication including several teratogenic drugs. Thirty-five pregnancies terminated in live births without congenital abnormalities, 3 in live births with congenital anomalies, 23 in miscarriages, 2 in stillbirths without congenital anomaly, 20 were electively terminated, and 12 ongoing/unknown. Of the three congenital anomalies, one was due to Dandy–Walker syndrome, one was associated with the exposure to a known teratogenic drug, and the third was due to a chromosomal translocation in the mother and could not be attributed to belimumab [88]. The British Society of Rheumatology suggests that belimumab should not be administered during pregnancy and lactation [84]. In conclusion, there is insufficient human data to demonstrate the safety of belimumab use during pregnancy. 

## 8. Proteasome Inhibitors

### Bortezomib

The proteasome regulates protein expression and function by degradation of ubiquitylated proteins, and also cleanses the cell of abnormal or misfolded proteins. Proteasome inhibition may prevent degradation of pro-apoptotic factors, thereby triggering programmed cell death in neoplastic cells. Bortezomib is an N-protected dipeptide that binds the catalytic site of the 26S proteasome with high affinity and specificity. It causes a rapid and dramatic change in the levels of intracellular peptides that are produced by the proteasome [89]. Bortezomib is approved for treating patients with multiple myeloma and has been used also in patients with mantle lymphoma and some cases of antibody-mediated rejection.

Bortezomib is classified in category D by FDA. It can cause embryo-fetal lethality in animal studies at doses lower than the clinical dose. There are no controlled data in human pregnancy. Pregnancy is contraindicated, and effective contraception is recommended for women of childbearing potential. Multiple myeloma is extremely uncommon during pregnancy. Whenever possible bortezomib should be prescribed after delivery. A review of 32 cases of pregnancy in women with multiple myeloma reported that cesarean section was the most common form of delivery (82%). About 88% of newborns were healthy, although 73% of them were premature, in part because of accelerated delivery in critical pregnancies [90]. The excretion of Bortezomib in breast milk is unknown.

## 9. Tyrosine Kinase Inhibitors (TKIs)

Tyrosine kinases are enzymes responsible for the activation of many proteins by signal transduction cascades. The proteins are activated by adding a phosphate group to the protein (phosphorylation), a step that TKIs inhibit. 

Imatinib mesylate is the progenitor of TKIs. It is approved for treatment of chronic myeloid leukemia, gastrointestinal stromal tumors, and some brain tumors. The drug may also exert favorable effects in some rheumatic diseases. It is available in the form of rigid capsules that should be taken in a single daily administration during a meal with a glass of water. Imatinib has been found to be teratogenic in rats and is not recommended for use during pregnancy. There is a paucity of data regarding patients on imatinib mesylate becoming pregnant and completing pregnancy. Also, the placental transfer of TKIs in humans is poorly understood. A study reported outcome data for 125 pregnancies exposed to imatinib. Of those, 50% delivered normal infants and 28% underwent elective terminations, three following the identification of abnormalities. There were a total of 12 infants in whom abnormalities were identified, three of which had strikingly similar complex malformations that are clearly a cause for concern. It appears that although most pregnancies exposed to imatinib are likely to have a successful outcome, there remains a risk that exposure may result in serious fetal malformations [91]. In another study, 22 cases of pregnancies in women with chronic myeloid leukemia under imatinib therapy were analyzed. Of them 9 women who planned pregnancy were in complete remission for at least 2 years and were advised to stop imatinib for 1 month prior to conception and 3 months after conception (first trimester). Willing male patients stopped therapy one month prior to conception of their wives. In 13 unplanned cases, all patients were in exposure to imatinib during conception. Twenty-two pregnancies resulted in seven male children and eight female children. There were three miscarriages and four elective abortions along with one case of hypospadias and another one of mild hydrocephalus, showing that unplanned pregnancy can result in miscarriage or congenital anomaly [92].

Second generation TKIs include dasatinib, nolitinib, and bovatinib. These TKIs work by blocking a number of tyrosine kinases such as the Bcr-Abl and the Src kinase family. They are used in the treatment of chronic myeloid leukemia and acute lymphoblastic leukemia positive for the Philadelphia chromosome. There are limited data on the use of dasatinib in pregnancy. In a review, 15 of 46 women (33%) delivered a normal infant; 18 (39%) and 8 (17%) had an elective or spontaneous abortion, and 5 (11%) had an abnormal pregnancy. There were 7 reports of fetal/infant abnormalities (encephalocele, renal tract abnormalities, and hydrops fetalis). Instead, 30 of 33 (91%) infants fathered by dasatinib-treated men were reported normal at birth [93]. In the nilotinib investigator’s brochure, there was only one case with fetal abnormalities out of 45 pregnancies exposed to the drug. In addition, there was one female exposed pregnant of twins with one twin experiencing congenital transposition of great vessels resulting in death, and the other twin experiencing a non-serious heart murmur. Women of child bearing potential with chronic myeloid leukemia should be informed about the risks of taking dasatinib during pregnancy [94]. Third-generation TKIs include erlotinib, gefitinib, bosutinib, and ponatinib. They are used in chronic myeloid leukemia and are the first-line targeted therapy in case of stage IV non-small-cell lung cancer. These drugs have a low transplacental transfer. Little is known about their use in pregnancy. Their teratogenicity seems to be similar to that observed with the use of other TKIs [95].

In view of the potential embriotoxic effects TKIs are classified in the category D by FDA. All TKIs should be avoided during the organogenesis. The safest potential therapeutic options for the management of chronic myeloid leukemia in pregnancy include temporary discontinuation of the tyrosine kinase inhibitor followed by observation or intervention with interferon-α and/or leucapheresis [96]. Therapy with TKI should be considered only if a cytogenetic or hematologic relapse occurs and no effective alternative treatments exist.

## 10. Fusion Proteins

Etanercept is a soluble fusion protein produced by recombinant DNA that binds specifically to TNF and acts as a TNF inhibitor. Etanercept mimics the inhibitory effects of soluble TNF receptors, the difference being that etanercept has an extended half-life and a more profound and long-lasting biologic effect than the natural soluble TNF receptor. Like anti-TNFα monoclonal antibodies, also Etanercept is used to treat rheumatoid arthritis, juvenile rheumatoid arthritis. Psoriatic arthritis, plaque psoriasis, and ankylosing spondylitis. A number of studies showed that etanercept may carry a risk of adverse pregnancy outcome of moderate clinical relevance, similar to that of TNFα antibodies [97,98].

Abatacept is a fusion protein composed of the Fc region of the immunoglobulin IgG1 fused to the extracellular domain of CTLA-4. It is used in rheumatoid arthritis and psoriatic arthritis. In a review 151 pregnancies following maternal exposure to abatacept were reported. Seven of 86 (8.1%) live births had congenital anomalies (cleft lip/cleft palate, congenital aortic anomaly, meningocele, pyloric stenosis, skull malformation, ventricular septal defect/congenital arterial malformation, and Down’s syndrome with premature rupture of membranes at 17 weeks that resulted in a live birth via cesarean section and subsequent infant death). In addition, 59 of the 151 (39.0%) cases with maternal exposure resulted in abortions (40 spontaneous and 19 elective). The investigators concluded that abatacept should be used during pregnancy only if the potential benefit to the mother justifies the potential risk to the fetus [99].

## 11. Hydroxychloroquine (HCQ)

HCQ is the hydroxylated analogue of chloroquine. The drug has been initially used as an antimalarial agent, since it may inhibit the plasmodial heme polymerase. HCQ is not classified among immunosuppressive drugs. However, HCQ can cause expansion and vacuolization of lysosomes and inhibition of their functions [100]. These changes can interfere with the function of the immunocompetent cells and cause downregulation of the immune response against auto-antigenic peptides, a property that can be exploited in the treatment of systemic lupus erythematosus.

Animal studies have revealed evidence of fetal harm. Use of chloroquine and derivates in high doses and for prolonged durations has been associated with neurological disturbances and interference with hearing, balance, and vision in the fetus. There are no controlled data in human pregnancy. HCQ is considered in category C by the FDA. Large series in pregnant patients with connective tissue diseases reported that the outcomes of pregnancy and fetal outcome were not different in women who received HCQ and controls with similar disorders [101,102]. In patients with SLE, the drug does not cause fetal toxicity, while the cessation of HCQ treatment during pregnancy increases the degree of lupus activity [103]. The beneficial effect of HCQ use during pregnancy in SLE is underscored by the significantly decreased rate of prematurity and intrauterine growth restriction in the vulnerable preterm birth population [104,105]. Infants exposed to hydroxychloroquine during breastfeeding receive only small amounts of the drug in breastmilk. In a small number of infants up to at least one year of age, careful follow-up found no adverse effects on growth, vision, or hearing [106,107]. International experts indicate that hydroxychloroquine is acceptable during breastfeeding [108,109].

On the basis of this data we feel that HCQ therapy may be continued throughout pregnancy in patients with SLE.

## 12. Methotrexate

Methotrexate is an antimetabolite of the antifolate type. Methotrexate competitively inhibits dihydrofolate reductase, an enzyme that catalyzes the conversion of dihydrofolate to the active tetrahydrofolate. Folic acid is needed for the *de novo* synthesis of the nucleoside thymidine, required for DNA synthesis. Also, folate is essential for purine and pyrimidine base biosynthesis, so synthesis will be inhibited. Methotrexate, therefore, inhibits the synthesis of DNA, RNA, thymidylates, and proteins [110]. Methotrexate is used in the chemotherapy of malignant tumors, such as choriocarcinoma and other trophoblastic tumors, and for the maintenance therapy of acute lymphocytic leukemia. The drug is also used for the treatment of patients with rheumatoid arthritis (and in several other forms of inflammatory arthritis and autoimmune disease.

Methotrexate causes teratogenic effects, embryotoxicity, abortion, and fetal defects in humans. It has also been reported to cause impairment of fertility, oligospermia, and menstrual dysfunction in humans, during and for a short period after cessation of therapy. The drug is classified in category X by the FDA and should not be used in pregnancy unless it is indicated to favor the termination of intrauterine or *ectopic* pregnancy [111].

## 13. Conclusions

Planning pregnancy during the quiescent phases of diseases is the prerequisite for a successful outcome for the mother and for the fetus. The treatment necessary to control the disease during pregnancy avoiding fetal risks is not a minor issue and currently it represents a real challenge. For a number of drugs on the market for many years, available data suggest that short-acting glucocorticoids, azathioprine and calcineurin inhibitors may be considered “relatively” safe for the risk of major congenital malformations. The introduction of a series of biological drugs in the treatment of many diseases has improved their prognosis, making it possible for a growing number of women to become pregnant. Unfortunately, few data are available about the compatibility of biologic drugs with reproduction. TNF inhibitor treatment has not been associated with an increased risk of malformations. These are the only biologic agents for which, when strictly indicated, the available data support the use at least in the first trimester of pregnancy. No other biologic drugs have been studied sufficiently during human pregnancy to draw firm conclusions about safety. While waiting for more robust data, it is recommendable to withdraw these drugs before conceiving.

Unfortunately, also data on the long-term safety in the antenatally exposed child are still lacking. This represents a major concern in view of reports outlining the association between pre-natal events and late development of neurological and metabolic disorders. Only large-scale population with long-term follow-up of the infants born to women taking a certain drug during pregnancy will allow to consider safe for the offspring that particular medicament. 

## Figures and Tables

**Table 1:** The food and drug administration classification of teratogenic drugs.

Category	Human Fetus Risk	Drugs
A	No fetal risk	
B	No risk of human fetus. Possible animal risk but human studies lacking.	Infliximab, Adalimumab, Golimumab, Certolizumab, Etanercept
C	Human risk cannot be ruled out	Glucocorticoids, Cyclosporine, Tacrolimus, Azathioprine, Sirolimus, Everolimus, Rituximab, Eculizumab, Belimumab, Hydroxychloroquine
D	Evidence of risk to human fetus	Mycophenolate, Cyclophos phamide, Chlorambucil, Bortezomib, Tyosine-kinase inhibitors
X	Contraindication in pregnancy	Leflunomide, Methotrexate
